# SARA-GAN: Self-Attention and Relative Average Discriminator Based Generative Adversarial Networks for Fast Compressed Sensing MRI Reconstruction

**DOI:** 10.3389/fninf.2020.611666

**Published:** 2020-11-26

**Authors:** Zhenmou Yuan, Mingfeng Jiang, Yaming Wang, Bo Wei, Yongming Li, Pin Wang, Wade Menpes-Smith, Zhangming Niu, Guang Yang

**Affiliations:** ^1^School of Information Science and Technology, Zhejiang Sci-Tech University, Hangzhou, China; ^2^College of Communication Engineering, Chongqing University, Chongqing, China; ^3^Aladdin Healthcare Technologies Ltd., London, United Kingdom; ^4^Cardiovascular Research Centre, Royal Brompton Hospital, London, United Kingdom; ^5^National Heart and Lung Institute, Imperial College London, London, United Kingdom

**Keywords:** MRI, reconstruction, deep learning, compressive sensing, neuroinformatics, artificial intelligence, GAN

## Abstract

Research on undersampled magnetic resonance image (MRI) reconstruction can increase the speed of MRI imaging and reduce patient suffering. In this paper, an undersampled MRI reconstruction method based on Generative Adversarial Networks with the Self-Attention mechanism and the Relative Average discriminator (SARA-GAN) is proposed. In our SARA-GAN, the relative average discriminator theory is applied to make full use of the prior knowledge, in which half of the input data of the discriminator is true and half is fake. At the same time, a self-attention mechanism is incorporated into the high-layer of the generator to build long-range dependence of the image, which can overcome the problem of limited convolution kernel size. Besides, spectral normalization is employed to stabilize the training process. Compared with three widely used GAN-based MRI reconstruction methods, i.e., DAGAN, DAWGAN, and DAWGAN-GP, the proposed method can obtain a higher peak signal-to-noise ratio (PSNR) and structural similarity index measure(SSIM), and the details of the reconstructed image are more abundant and more realistic for further clinical scrutinization and diagnostic tasks.

## Introduction

MRI can carry out the non-invasive examination of the internal tissues of the human body, so it is widely used in clinical pathological examination and diagnosis (Liang and Lauterbur, 2000; Kabasawa, 2012). However, the excessive scanning time of MRI limits its clinical application, and this problem is particularly prominent for high-resolution imaging. Therefore, how to reduce k-space sampling (Duyn et al., 1998) and shorten MRI acquisition time has become a research focus in this field. Compressed sensing (CS) (Lustig et al., 2008, 2010) is a conventional method for solving this problem, it uses the compressibility and sparsity of the signal to reduce k-space sampling and achieve fast imaging. At present, the methods surrounding compressed sensing for fast MRI mainly include non-Cartesian CS (Haldar et al., 2011; Wang et al., 2012), combination parallel imaging with CS (Murphy et al., 2012; El Gueddari et al., 2019; Shimron et al., 2020), and CS-based on dictionary learning (Ravishankar and Bresler, 2010; Huang et al., 2014; Du et al., 2019; Cao et al., 2020). Although the above-mentioned methods based on compressed sensing have achieved good results, they all rely on the prior knowledge extracted from the image, which limits the use of the above methods to a certain extent.

In recent years, deep learning (LeCun et al., 2015) methods have been successfully applied in many fields. In particular, with the emergence of convolutional neural networks, made it show great performance in computer vision. At present, many MRI reconstruction methods based on deep learning have been proposed (Boyd et al., 2011; Sun et al., 2016; Wang et al., 2016; Aggarwal et al., 2018; Zhu et al., 2018; Akçakaya et al., 2019; Lee et al., 2020). In 2016, Wang et al. (2016) first applied deep learning methods to the acceleration of MRI imaging. They employed an offline CNN to realize the mapping of undersampled zero-filled MRI and fully sampled k-space data and achieved good reconstruction effectively. Deep learning based MRI reconstruction methods can be roughly divided into unrolling-based approaches and those not based on unrolling (Liang et al., 2020). Among them, the unrolling-based method usually expands the CS-based iterative reconstruction algorithm into a deep network, so that the parameters in the reconstruction algorithm can be learned by the network. Sun et al. (2016) proposed the ADMM-Net defined over data flow graphs, which were derived from the iterative procedures in the Alternating Direction Method of Multipliers (ADMM) algorithm (Boyd et al., 2011) for optimizing a general CS-based MRI mode, and it significantly improved the baseline ADMM algorithm and achieved high reconstruction accuracies with fast computational speed. The framework proposed by Aggarwal et al. (2018), termed as MOdel-based reconstruction using Deep Learned priors (MODL), merged the power of model-based reconstruction schemes with deep learning. Their model provided improved results, despite the relatively smaller number of trainable parameters. The methods not unrolling-based mainly uses deep networks to learn the mapping between under-sampled data and fully sampled data to achieve reconstruction. Zhu et al. (2018) proposed a unified framework—automated transform by manifold approximation (AUTOMAP), it constructed a supervised learning task to learn the mapping between sensor domain and image domain from training data. Besides, Robust artificial-neural-networks for k-space interpolation (RAKI) (Akçakaya et al., 2019) was proposed for image reconstruction by training convolutional neural networks on ACS data. Compared with the traditional linear k-space interpolation-based method, this method had better anti-noise performance.

The Generative Adversarial Networks (GAN) (Goodfellow et al., 2014) proposed by Goodfellow was a novel deep generative model, which introduced the idea of game theory and improved the fitting ability of the network through the competitive learning of generator and discriminator. In 2016, Radford et al. proposed Deep Convolutional Generative Adversarial Networks (DCGAN) to apply convolutional neural networks to unsupervised learning (Radford et al., 2016). By applying convolutional neural networks to generators and discriminators, the network could learn a hierarchy of representations from object parts to scenes. At present, GAN and its variants have achieved excellent performance in image-to-image translation (Zhu et al., 2017), image super-resolution (Ledig et al., 2017), and others. In recent years, since its good data representation capabilities, GAN have also been used for MRI fast imaging (Arjovsky et al., 2017; Yang et al., 2017; Jiang et al., 2019; Kwon et al., 2019) and super-resolution (Chen et al., 2018; Lyu et al., 2019; Mahapatra et al., 2019). Yang et al. (2017) applied conditional GAN to MRI reconstruction and proposed the De-Aliasing Generative Adversarial Networks (DAGAN) model. Compared with conventional methods, the DAGAN model achieved a better reconstruction effect and retained more perceptible details. Wasserstein GAN (Arjovsky et al., 2017) is a variant of the original GAN, by replacing the Jensen-Shannon divergence in the original GAN with Wasserstein distance, it stabilizes the learning process and solves the problem of mode collapse. Jiang et al. (2019) proposed a de-aliasing fine-tuning Wasserstein generative adversarial network (DA-FWGAN) for MR imaging reconstruction. The DA-FWGAN could provide reconstruction with improved peak signal-to-noise ratio (PSNR) and structural similarity index measure (SSIM).

Although the current MRI reconstruction methods based on deep learning can better learn the mapping relationship between undersampling MRI and full sampling MRI, the reconstruction effect still has a lot of room for improvement. Firstly, most GANs use convolutional layers to build their generators. Due to the limited size of the convolution kernel, the network can only focus on the dependencies of the information in the local receptive field (Luo et al., 2016), but it cannot establish the long-range dependencies of the image, which leads to the inaccurate reconstruction of the image details and texture. Self-Attention Generative Adversarial Networks (SA-GAN) (Zhang et al., 2019) proposed by Zhang et al. solved this problem by introducing a self-attention mechanism and constructing long-range dependency modeling. The self-attention mechanism was used for establishing the long-range dependence relationship between the image regions. To enhance the image details and improve the quality of reconstructed MRI, the local dependence, and the global dependence of the image were combined. Secondly, the discriminator did not make full use of the prior knowledge that half of the input data is true and half is fake (Jolicoeur-Martineau, 2018). When the generated data is real enough, the discriminator can directly distinguish the generated data into real data, which results in the insufficient performance of the discriminator and the training of the generator cannot be continued. Alexia Jolicoeur-Martineau used the prior knowledge to induce a “relative discriminator” (Jolicoeur-Martineau, 2018), which estimated the probability that the given real data was more realistic than a randomly sampled fake date.

In this paper, we propose a novel MRI reconstruction method, termed as SARA-GAN, which combines the self-attention mechanism and the relative discriminator. The generator is designed as a structure, composing of down-sampling block, residual block, and up-sampling block. Among them, in the up-sampling block, we add a self-attention layer to capture the global information of the image. Besides, the discriminator uses the CNN structure and introduces the idea of relative discrimination to construct a relative average discriminator. At the same time, we also apply spectral normalization on the generator and discriminator to stabilize the training process. The novelties of our proposed SARA-GAN model have been summarized as follows

- Given the traditional convolutional structure that can only focus on the local dependency of the image, we add a self-attention layer to the high-layer of the generator network. The self-attention mechanism can calculate the correlation degree between image pixels and build long-range dependencies so that the reconstructed image can demonstrate more details.
- The theoretical formula of the original GAN-based methods ignores the prior information of the discriminator's input data. In our SARA-GAN model, we use relative average discriminator to transform the absolute true or false discrimination into relative true or false. In doing so, our SARA-GAN model can make full use of the prior information, and therefore can improve the discriminator performance.
- In our SARA-GAN, the generator adopts a residual network structure, in which multiple residual blocks are cascaded and multiple skip connections are incorporated to reduce the loss of original features in the convolution calculation. At the same time, this can avoid poor performance of the generator in the initial training stage; therefore, the training procedure can be more efficient.
- We also apply the spectral normalization to the network parameters of the generator and the discriminator to satisfy the Lipschitz constraint, thereby stabilizing the training of our GAN-based SARA-GAN model.

## Methods

**Figure 1:**
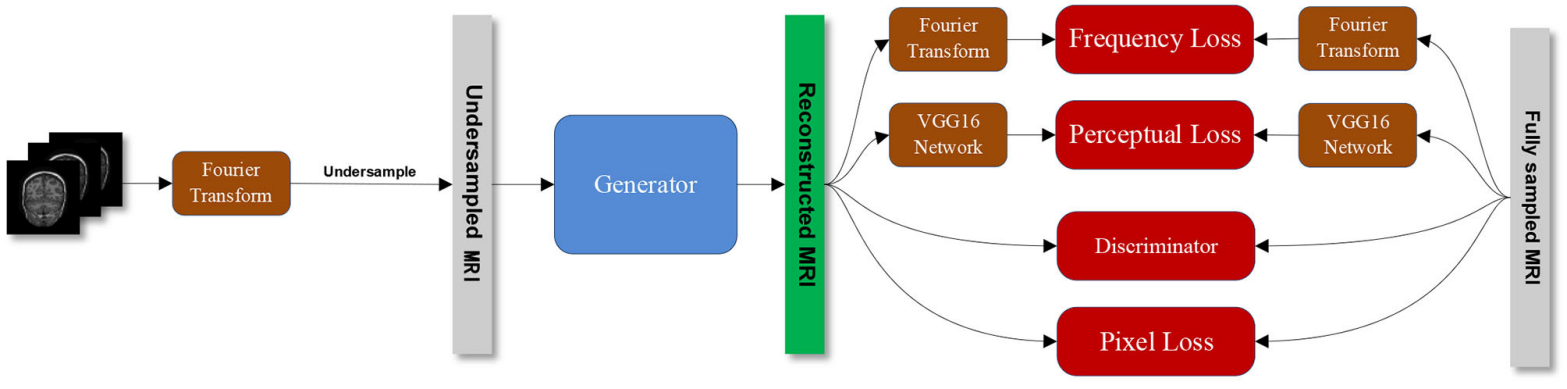
The overall structure of the proposed SARA-GAN method.

Figure 1 shows the overall structure of our proposed SARA-GAN. We obtain the k-space data of the fully sampled MRI through Fourier transform, then undersampled the k-space data, and perform inverse Fourier transform to obtain the image-domain undersampled MRI. The generator is used to learn the mapping relationship between undersampled MRI and full-sampled MRI. The discriminator is a binary classifier, used to judge whether the reconstructed image is true or false. The combined loss function incorporates the pixel loss, the perceptual loss, and the frequency-domain loss based on the adversarial loss. The pixel loss and the perceptual loss can constrain GAN training on the image content. The frequency-domain loss provides additional constraints for the data consistency in the k-space. The pre-trained VGG16 network is used to extract features from the fully sampled MRI and the reconstructed MRI respectively, and the two sets of features are compared to obtain the perception loss. The discriminator and the combined loss function guide the training of the generator together.

### Network Structure

#### Generator Model

**Figure 2:**
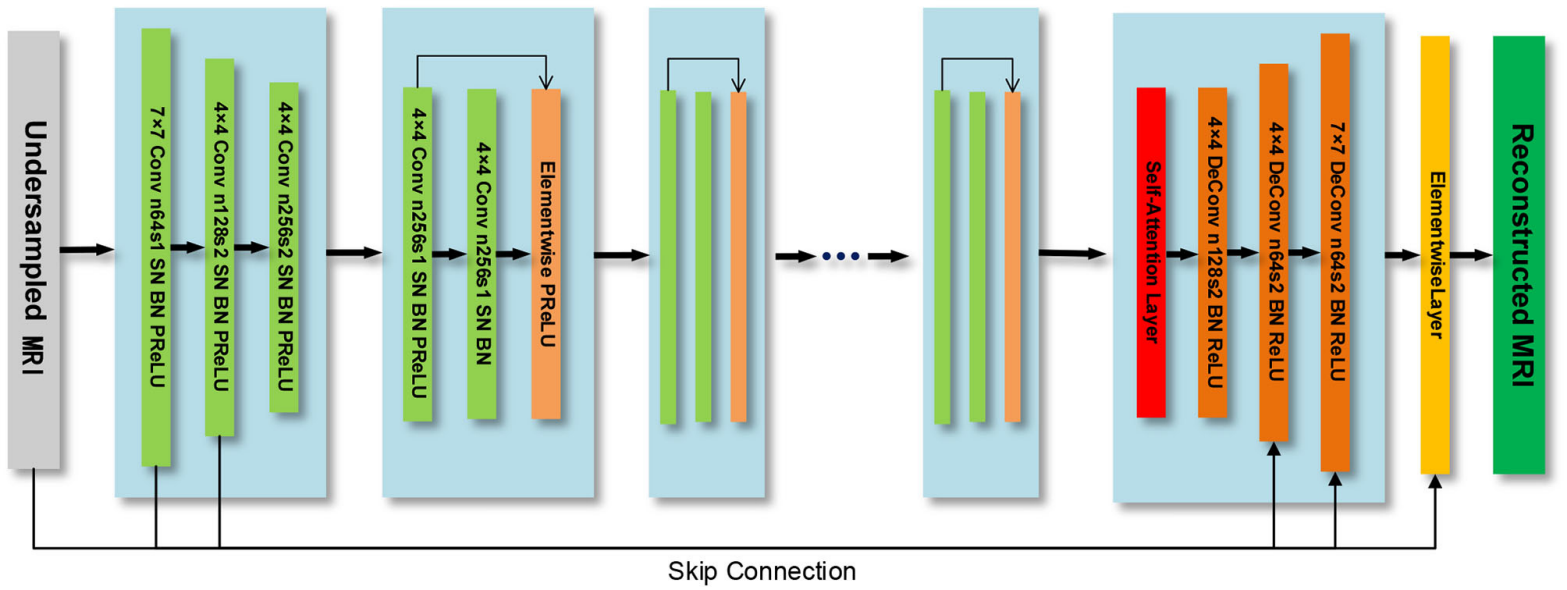
The generator model.

The generator model is composed of a down-sampling block, residual block, and up-sampling block. The three convolutional layers in the down-sampling block are used to extract image features. The residual block contains 7 residual blocks, and each residual block contains two convolutional layers. The up-sampling block consists of three transposed convolutional layers, which are used to expand the feature map and generate reconstructed MRI. We use spectral normalization on the generator network and choose the PReLU (He et al., 2015) function as the activation function. Besides, we introduce the self-attention module in the up-sampling block to build the long-range dependency of the image, as shown in Figure 2.

#### Discriminator Model

**Figure 3:**
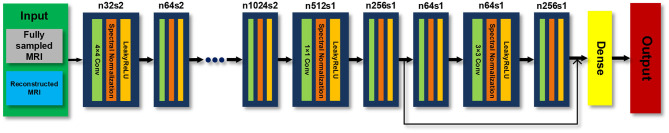
Discriminator model.

The discriminator model is an 11-layer CNN network, which uses leaky ReLU as the activation function. The last layer is the dense layer, and the sigmoid function is used as the activation function to output the discriminatory results of the discriminator, as shown in Figure 3. We also use spectral normalization in the discriminator.

### Self-Attention Module

To overcome the problem that the network cannot learn long-range global dependencies caused by the limited size of the convolution kernel, we add the self-attention (Zhang et al., 2019) into the up-sampling block of the generator, as shown in Figure 2. In the self-attention module, the output feature map of the last residual block *x* with the channel number *C* of the previous convolution layer is input to three convolution layers with a kernel of 1 × 1 and the channel numbers of *C*/8, *C*/8 and *C* respectively, to obtain the feature space *f*(*x*),*g*(*x*) and *h*(*x*)

(1) $$\begin{array}{l} f\left(x\right)=W_{f}x, \end{array}$$

(2) $$\begin{array}{l} g\left(x\right)=W_{g}x, \end{array}$$

(3) $$\begin{array}{l} h\left(x\right)=W_{h}x. \end{array}$$

Then the transpose of *f*(*x*_*i*_) is multiplied by *g*(*x*_*j*_), and the weight is normalized by the Softmax function to obtain β_*j,i*_

(4) $$\begin{array}{l} s_{ij}=f{\left(x_{i}\right)}^{T}g\left(x_{j}\right), \end{array}$$

(5) $$\begin{array}{l} \beta_{j,i}=\frac{\exp\left(s_{ij}\right)}{\overset{N}{\underset{i=1}{\sum }}\exp\left(s_{ij}\right)}, \end{array}$$

where β_*j,i*_ is an attention map that indicates the extent to which the model attends to the *i*^*th*^ location when synthesizing the *j*^*th*^ region. The output of the self-attention layer is defined as

(6) $$\begin{array}{l} o_{j}=v\left[\overset{N}{\underset{i=1}{\sum }}\beta_{j,i}h\left(x_{i}\right)\right],v\left(x_{i}\right)=W_{v}x_{i} \end{array}$$

In the above formula, *W*_*f*_, *W*_*g*_,*W*_*h*_, and *W*_*v*_ are the weight matrices of the 1 × 1 convolutional layer. To allow the generator learns the local dependence of the image as well as the long-range global dependence, we multiply the output of the self-attention layer *o*_*j*_ by a weight coefficient γ and add it to the input feature map *x*_*i*_ to obtain the final output of the self-attention module *y*_*i*_

(7) $$\begin{array}{l} y_{i}=\gamma o_{i}+x_{i}. \end{array}$$

Among them, γ is a learnable parameter and is initialized to 0. Its function is to enable the network to learn the proportion of the global dependency on the feature map.

### Relative Average Discriminator

In the original GAN model, the generator accepts random noise, and then generates a false image and inputs it to the discriminator. The discriminator gives the probability that the input image belongs to the real image. The two compete with each other and learn together. Finally, the generator learns the probability distribution of the real image, making the discriminator unable to distinguish between the real image and the generated image, and then achieves Nash equilibrium.

Specifically, in the problem of MRI image reconstruction, *x* is defined as the fully sampled MRI image, and *z* is the undersampled zero-filled MRI image. The theoretical formula of the original GAN is:

(8) $$\begin{array}{l} \max L_{D}={\text{E}}_{x\sim \;P_{\text{data}}\left(x\right)}\left[\log D\left(x\right)\right]\\ \;+{\text{E}}_{z\sim \;P_{z}\left(z\right)}\left[\log\left(1-D\left(G\left(z\right)\right)\right)\right], \end{array}$$

(9) $$\begin{array}{l} \min L_{G}={\text{E}}_{z\sim \;P_{z}\left(z\right)}\left[\log\left(1-D\left(G\left(z\right)\right)\right)\right], \end{array}$$

where *P*_data_(*x*) is the fully sampled MRI image distribution, *P*_*z*_(*z*) is the undersampled zero-filled MRI image distribution. The optimization process of the original GAN is essentially to reduce the Jensen–Shannon divergence (JSD) between *P*_data_(*x*) and *P*_*z*_(*z*)

(10) $$\begin{array}{l} JSD(P_{data}\|P_{z})=\frac{1}{2}(\text{log}(4)+\underset{D}{\text{max }}{\text{E}}_{x\sim P_{data}(x)}[\text{log}D(x)]\\ \;+{\text{E}}_{z\sim P_{z}(z)}[\text{log}(1-D(G(z)))]). \end{array}$$

When $D\left(x\right)\text{=}D\left(G\left(z\right)\right)\text{=}\frac{\text{1}}{\text{2}}$, *JSD*(*P*_*data*_‖*P*_*z*_) gets the minimum value 0. Therefore, ideally, when the generator generates sufficiently real samples, the discriminator cannot distinguish between true and false samples and should output a probability value of 0.5. However, in actual training, the above formula may cause the expected output of the discriminator D to be 1. This is because the original GAN theoretical formula ignores a priori knowledge, for instance, in a minibatch, half of the samples' input to the discriminator are real data and the other half are generated data.

We use the relative average discriminator (Jolicoeur-Martineau, 2018) and believe that the discriminator should estimate the probability that the given full sampling MRI is more realistic than the reconstruction MRI, on average, by making full use of the above prior knowledge. Therefore, the theoretical formula after using the relative average discriminator in our work is

(11) $$\begin{array}{l} \min L_{D}=-{\text{E}}_{x\sim P_{data}}\left[\log\left(D\left(x\right)\right)\right]\\ \;-{\text{E}}_{z\sim P_{z}}[\log\left(1-D\left(G\left(z\right)\right)\right], \end{array}$$

(12) $$\begin{array}{l} \min L_{G}=-{\text{E}}_{z\sim P_{z}}\left[\log\left(D\left(G\left(z\right)\right)\right)\right]\\ \;-{\text{E}}_{x\sim P_{data}}\left[\log\left(1-D\left(x\right)\right)\right], \end{array}$$

(13) $$\begin{array}{l} D\left(x\right)=sigmoid\left(C\left(x\right)-{\text{E}}_{z\sim P_{z}}C\left(G\left(z\right)\right)\right)\\ D\left(G\left(z\right)\right)=sigmoid\left(C\left(G\left(z\right)\right)-{\text{E}}_{x\sim P_{data}}C\left(x\right)\right), \end{array}$$

where *C*(·) is the output of the discriminator network.

### Spectral Normalization

Miyato et al. (2018) proposed to apply spectral normalization (SN) to the discriminator network to stabilize GAN training. In this study, we also use spectral normalization in the weights of the generator network and discriminator network. The spectral normalization method uses the spectral norm on the parameter matrix of the discriminator and generator network, so that the network satisfies the Lipschitz constraint, thereby smoothing the network parameter to stabilize training.

### Loss Function

The loss function is used to evaluate the gap between the reconstructed image and the fully sampled image, which is the optimization object of the GAN. The smaller the loss function value, the smaller the gap between the reconstructed image and the fully sampled image, and the better the reconstruction effect. A reasonable loss function can provide accurate gradient information for network training, thereby improving reconstruction performance. We use a combined loss function that combines perceptual loss, pixel loss, frequency domain loss, and adversarial loss to comprehensively evaluate the fitting ability of the network.

The pixel loss *L*_*pixel*_ and frequency domain loss *L*_*frequency*_ are based on Mean Square Error(MSE), can be defined as follows

(14) $$\begin{array}{l} \underset{G}{\min}L_{pixel}\left(G\right)=\frac{1}{2}{\left\|{\text{x}}_{\text{t}}-{\text{x}}_{\text{u}}\right\|}_{2}^{2}, \end{array}$$

(15) $$\begin{array}{l} \underset{G}{\min}L_{frequency}\left(G\right)=\frac{1}{2}{\left\|{\text{y}}_{\text{t}}-{\text{y}}_{\text{u}}\right\|}_{2}^{2}, \end{array}$$

where **x**_**t**_ and **x**_**u**_ are fully sampled and reconstructed MR images in the image domain, respectively. **y**_**t**_ and **y**_**u**_ correspond to the frequency domain data of **x**_**t**_ and **x**_**u**_, respectively. The perceptual loss and adversarial loss are defined as

(16) $$\begin{array}{l} \underset{G}{\min}L_{perceptual}\left(G\right)=\frac{1}{2}{\left\|f_{VGG16}\left({\text{x}}_{\text{t}}\right)-f_{VGG16}\left({\text{x}}_{\text{u}}\right)\right\|}_{2}^{2}, \end{array}$$

(17) $$\begin{array}{l} \underset{G}{\min}\;L_{adversarial}=-{\text{E}}_{z\sim p_{z}}\left[\log\left(D(G(z))\right)\right]\\ \;-{\text{E}}_{x\sim p_{data}}\left[\log\left(1-D(x)\right)\right], \end{array}$$

where *f*_*VGG*16_ represents the VGG16 network (Russakovsky et al., 2015), *D*(·) represents the relative average discriminator.

Therefore, the final total loss function can be expressed as

(18) $$\begin{array}{l} L_{total}=\alpha L_{pixel}+\beta L_{frequncy}+\gamma L_{perceptual}+L_{adversarial}, \end{array}$$

where α, β and γ are the weight parameter of each loss function.

## Experiments

### Experimental Setup

**Figure 4:**
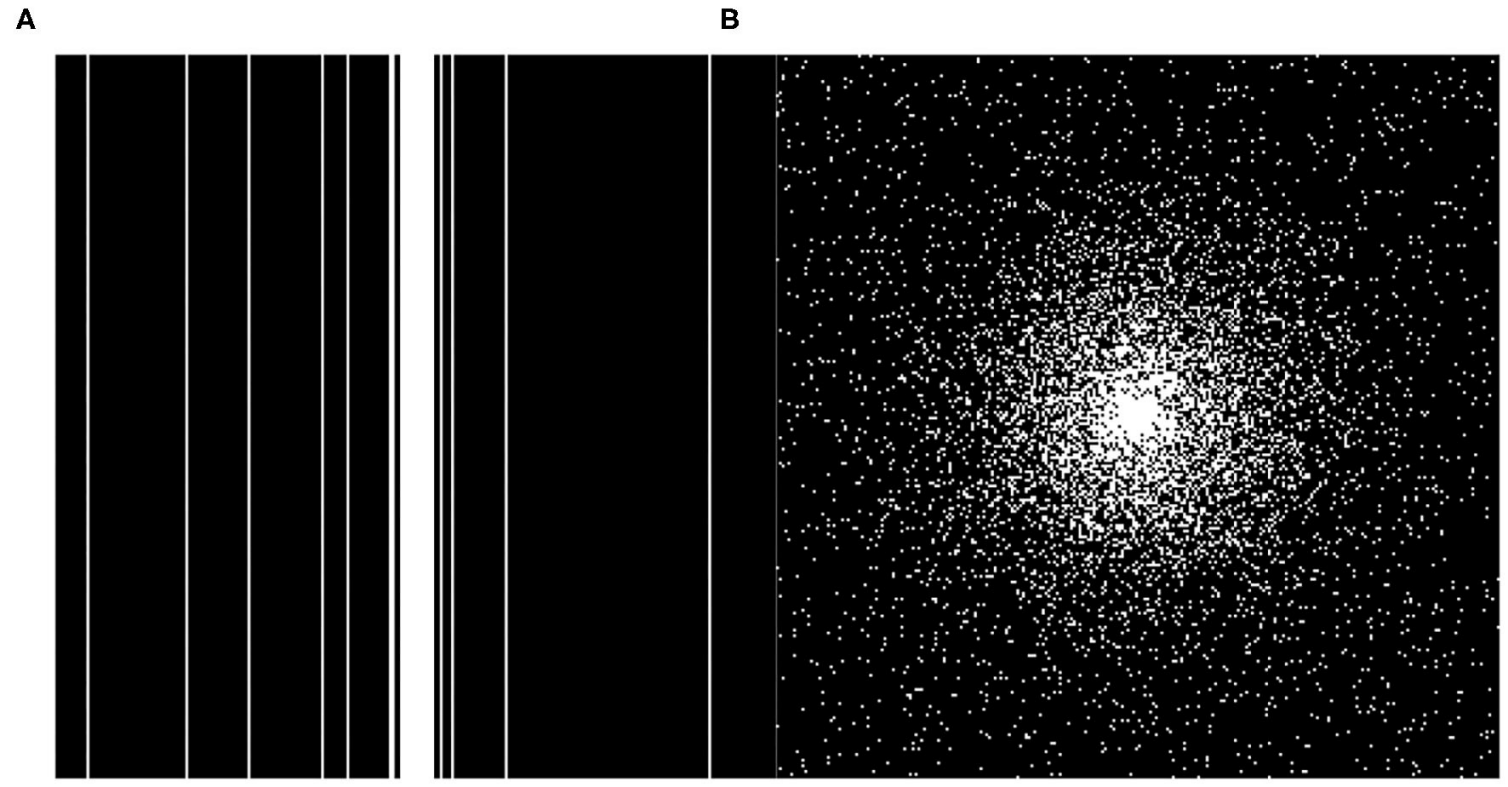
The two different under sampling masks. **(A)** 1D Gaussian mask and **(B)** 2D Gaussian mask.

The datasets used in this article are downloaded from the Diencephalon Challenge (https://www.synapse.org/#!Synapse:syn3193805/wiki/217780) in the public repository of the MICCAI 2013 grand challenge. The MRI data acquisition method is MPRAGE, the scanning matrix size is 256 × 256 × 287, and the resolution is 1 × 1 × 1 mm. We randomly selected 130 3D neuro-MRI images from the data set to verify the proposed SARA-GAN model. In the experiments, 70 samples (15,816 effective 2D MRIs) were used as the training set, 30 samples (5,073 effective 2D MRIs) were used as the validation set, and 30 samples (5,198 effective 2D MRIs) were used as the test set. In order to enhance the network performance, we applied data augmentation to the training dataset, including flipping (left to right), rotating ±20 degrees, shifting 10% along the x-axis and y-axis, random zooming between 0.9 times and 1.1 times, random brightness changes, and the random elastic transformation with alpha of 255 × 3 and sigma of 255 × 0.10. We use TensorFlow 1.12.0 and Python framework to program, and train the proposed model on a TeslaV100-SXM2 GPU under a CentOS system environment. Two undersampling modes, including 1-dimensional Gaussian distribution and 2-dimensional Gaussian distribution, three sampling rates of 10, 20, and 30% (Corresponding to 10×, 5×, and 3.3× acceleration factors respectively) were used for obtaining undersampling MRI. We train the model separately for each sampling mode. The sampling modes are shown in Figure 4. The contrast experiments were carried out under the above conditions.

The input and output image size of the generator is 256 × 256, batch size set to 16. We set the learning rate of the generator and the discriminator to 0.0001 and 0.0002, respectively, so that the generator and the discriminator can learn simultaneously. Since loss items in the combined loss are inconsistent on the number scale; therefore, we use hyperparameters α, β and γ to balance them into a similar scale to make the final loss function more accurate. The hyperparameters α, β, and γ in the combined loss function are set to 15, 0.1, and 0.0025, respectively. The choice of these hyperparameters were tuned empirically for better reconstruction performance.

We use the Adam optimizer with Gradient Centralization (Yong et al., 2020) to optimize the loss function, and set the exponential decay rate for the 1st moment estimates (β_1_) to 0.5, and the exponential decay rate for the 2nd moment estimates (β_2_) to 0.999. To prevent over-fitting, we use the normalized mean square error (NMES) as an indicator to evaluate the fitting effect of the network on the validation set every epoch. After the network is trained for 30 epochs, the training is terminated, and the optimal model with the smallest NMSE is saved.

### Reconstruction Quality Evaluation

In our experiment, the peak signal-to-noise ratio (PSNR) and structural similarity index measure (SSIM) were used as evaluation indexes of the reconstructed image. PSNR and SSIM are defined as following

(19) $$\begin{array}{l} PSNR=10\log_{10}\left(\frac{255^{2}}{MN\overset{M}{\underset{i=1}{\sum }}\overset{N}{\underset{j=1}{\sum }}{\left(y_{i,j}-x_{i,j}\right)}^{2}}\right), \end{array}$$

where *x* represents the full sampling MRI, *y* represents the network reconstructed MRI, *i* and *j* represent the coordinates of image pixels, and *M, N* represents the size of the image.

(20) $$\begin{array}{l} SSIM=\frac{\left(2\mu_{x}\mu_{y}+C_{1}\right)\left(2\sigma_{xy}+C_{2}\right)}{\left(\mu_{x}^{2}+\mu_{y}^{2}+C_{1}\right)\left(\sigma_{x}^{2}+\sigma_{y}^{2}+C_{2}\right)} \end{array}$$

where μ_*x*_ and μ_*y*_ represent the means of image *x* and *y*, σ_*x*_ and σ_*y*_ represent the variances of image *x* and *y*, respectively.

## Results

**Figure 5:**
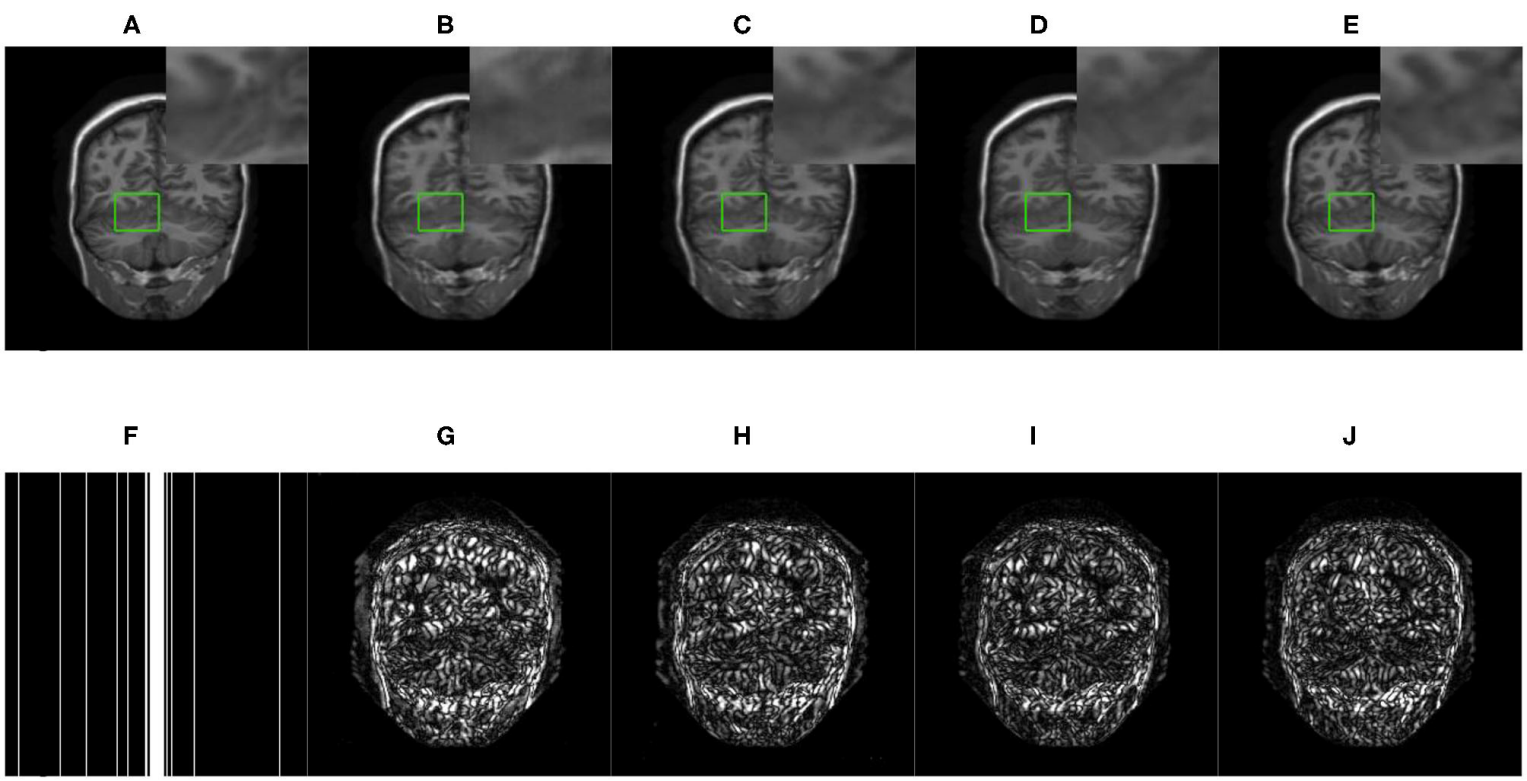
The reconstructed MRI for the 10-fold accelerated k-space data masked with the 1D Gaussian distribution by using different GAN-based methods. **(A)** Fully-sampled MRI, **(B)** DAGAN, **(C)** DAWGAN, **(D)** DAWGAN-GP, **(E)** SARA-GAN, **(F)** 1D mask, **(G)** DAGAN(error), **(H)** DAWGAN(error), **(I)** DAWGAN-GP(error), and **(J)** SARA-GAN(error).

**Figure 6:**
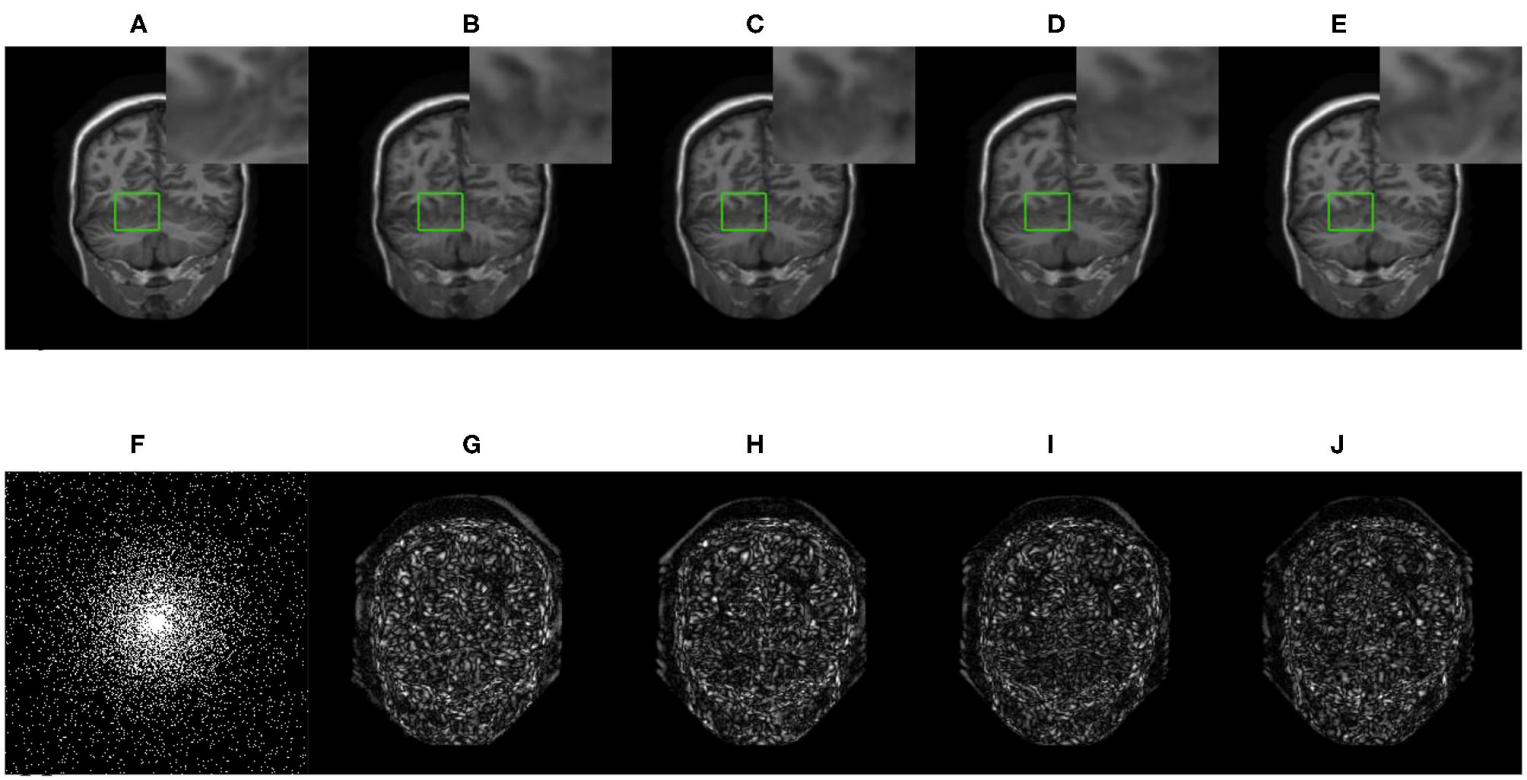
The reconstructed MRI for the 10-fold accelerated k-space data masked with the 2D Gaussian distribution by using different GAN-based methods. **(A)** Fully-sampled MRI, **(B)** DAGAN, **(C)** DAWGAN, **(D)** DAWGAN-GP, **(E)** SARA-GAN, **(F)** 2D mask, **(G)** DAGAN(error), **(H)** DAWGAN(error), **(I)** DAWGAN-GP(error), and **(J)** SARA-GAN(error).

We compared three GAN-based MRI reconstruction models, i.e., DAGAN, DAWGAN, DAWGAN-GP, and the compared methods all used the best parameter settings. Figures 5, 6 show the reconstruction effect of a typical MRI for the 10-fold accelerated k-space data masked with the Gaussian distribution using a different method. We chose to zoom in on a specific area of the MRI to compare the reconstruction details. From the local enlarged image, we can conclude that the reconstructed image obtained by the DAGAN method loses most of the texture information. DAWGAN and DAWGAN-GP perform slightly better than DAGAN, but there is still a big gap compared with full sampling MRI. Compared with the other three GAN-based methods, our method can restore more texture details, and the texture edge is clearer. The second line of Figures 5, 6 shows the reconstruction error map of different algorithms, and the color of pixels indicates the reconstruction error of the corresponding position. It can be seen that the reconstruction error of our method is less than that of other methods, indicating that the reconstructed MRI is closer to the full sampling MRI. Table 1 shows the quantitative comparison of the reconstruction effects of different methods. We calculate the average PSNR and SSIM of each method on the test set to evaluate the reconstruction performance of the model. Except for the first row in the table, our results are close to the DAWGAN-GP method. In the other undersampling modes, our method obtains higher PSNR and SSIM. The average PSNR is improved 0.04 dB ~ 0.96 dB over the DAWGAN-GP and the corresponding SSIM improvements are 0.0003 ~ 0.0008. In order to illustrate the performance of the proposed method, we estimate the statistical significancy using the Wilcoxon rank sum test (*p* < 0.05 indicates the significant difference). We find that except for 10% 2D Gaussian sampling experiment we have a similar performance between DAWGAN-GP and SARA-GAN (*p* = 0.1849), other experiments have demonstrated that our SARA-GAN has outperformed other methods significantly (most *p*-values are < 0.001).

**Table 1:** The average reconstruction performances of different methods on the test set (mean ± std).

	**DAGAN**		**DAWGAN**		**DAWGAN-GP**		**SARA-GAN**	
**Mask: 1D Gaussian**	**PSNR**	**SSIM**	**PSNR**	**SSIM**	**PSNR**	**SSIM**	**PSNR**	**SSIM**
Sample rate: 10%	34.0484 ± 4.81	0.9538 ± 2.20e-2	35.4455 ± 4.57	0.9654 ± 1.71e-2	36.3499 ± **4.27**	**0.9718** ± **1.37e-2**	**36.3926** ± 4.73	0.9713 ± 1.46e-2
Sample rate: 20%	40.2395 ± 4.23	0.9857 ± 6.41e-3	41.4284 ± 4.45	0.9894 ± 5.18e-3	42.6101 ± **3.92**	0.9923 ± 3.77e-3	**43.2054** ± 4.49	**0.9929** ± **3.71e-3**
Sample rate: 30%	40.8891 ± 4.46	0.9873 ± 5.88e-3	42.1798 ± 4.61	0.9907 ± 4.53e-3	42.9149 ± **3.69**	0.9928 ± **3.41e-3**	**43.3522** ± 4.34	**0.9931** ± 3.47e-3
**Mask: 2D Gaussian**	**PSNR**	**SSIM**	**PSNR**	**SSIM**	**PSNR**	**SSIM**	**PSNR**	**SSIM**
Sample rate: 10%	39.7242 ± 5.01	0.9801 ± 1.06e-2	40.7623 ± 4.68	0.9861 ± 7.75e-3	41.1885 ± **4.43**	0.9876 ± 6.85e-3	**41.6323** ± 5.25	**0.9881** ± **6.83e-3**
Sample rate: 20%	41.5595 ± 4.93	0.9857 ± 7.74e-3	41.7733 ± 5.15	0.9880 ± 6.84e-3	42.9742 ± **4.61**	0.9912 ± 5.05e-3	**43.4991** ± 5.15	**0.9920** ± **4.84e-3**
Sample rate: 30%	44.3886 ± 5.01	0.9934 ± 3.95e-3	44.2812 ± 5.11	0.9932 ± 4.03e-3	44.7868 ± **3.86**	0.9947 ± **2.97e-3**	**45.7536** ± 4.99	**0.9951** ± 3.03e-3

*The bold value means that the experimental result value is the best*.

With the increase of the acceleration factor, the reconstruction effect of either method becomes worse. At the same time, the reconstruction effect of 2-dimensional Gaussian sampling mode is obviously better than that of 1-dimensional Gaussian sampling. This is because the brain MRI has fewer texture details than natural images. The main information of brain MRI is concentrated in the low-frequency part of k-space, and the Gaussian sampling mode happens to also mainly collects the low-frequency part. Therefore, with the increase of sampling rate and sampling dimension, the information of the low-frequency part is more collected, so the reconstruction effect is also improved.

The real MRI sampling process often contains random noise. To simulate the real scene and evaluate the anti-noise ability of the model, we added 30 and 40 dB Gaussian white noise to the test set MRI and retested the above methods. Tables 2, 3 respectively show the reconstruction results of different algorithms on the test set with 30 and 40 dB Gaussian white noise. It can be seen from the table that, the average PSNR is improved by 0.004 ~ 0.841 dB over the DAWGAN-GP and the corresponding SSIM improvements are about 0.0004 ~ 0.0008. Despite the addition of a certain intensity of noise, our method still obtains a good reconstruction effect and is better than other GAN-based methods. This shows that our method has good anti-noise performance and the potential for practical application.

**Table 2:** The average reconstruction performances of different methods on the test set with 30dB noise.

	**DAGAN**		**DAWGAN**		**DAWGAN-GP**		**Proposed**	
**Mask: 1D Gaussian**	**PSNR**	**SSIM**	**PSNR**	**SSIM**	**PSNR**	**SSIM**	**PSNR**	**SSIM**
Sample rate: 10%	33.4169	0.9364	34.8801	0.9582	35.0774	0.9450	**35.3201**	**0.9535**
Sample rate: 20%	38.9860	0.9742	39.2584	0.9600	40.5054	0.9805	**41.4684**	**0.9878**
Sample rate: 30%	38.0020	0.9279	39.4411	0.9579	40.7167	0.9825	**41.5242**	**0.9886**
**Mask: 2D Gaussian**	**PSNR**	**SSIM**	**PSNR**	**SSIM**	**PSNR**	**SSIM**	**PSNR**	**SSIM**
Sample rate: 10%	39.1818	0.9775	39.5594	0.9827	40.5227	0.9859	**40.5901**	**0.9861**
Sample rate: 20%	40.3423	0.9807	40.1344	0.9843	41.6388	0.9890	**41.7555**	**0.9895**
Sample rate: 30%	42.5157	0.9907	42.3948	0.9901	42.6534	0.9919	**43.0708**	**0.9922**

*The bold value means that the experimental result value is the best*.

**Table 3:** The average reconstruction performances of different methods on the test set with 40dB noise.

	**DAGAN**		**DAWGAN**		**DAWGAN-GP**		**Proposed**	
**Mask: 1D Gaussian**	**PSNR**	**SSIM**	**PSNR**	**SSIM**	**PSNR**	**SSIM**	**PSNR**	**SSIM**
Sample rate: 10%	33.9782	0.9527	35.3805	0.9648	36.2549	**0.9711**	**36.2590**	0.9703
Sample rate: 20%	40.0935	0.9849	41.3256	0.9880	42.3872	0.9918	**42.9586**	**0.9926**
Sample rate: 30%	40.3222	0.9828	41.6212	0.9878	42.6548	0.9924	**43.1355**	**0.9928**
**Mask: 2D Gaussian**	**PSNR**	**SSIM**	**PSNR**	**SSIM**	**PSNR**	**SSIM**	**PSNR**	**SSIM**
Sample rate: 10%	39.6494	0.9797	40.2391	0.9843	41.1332	0.9874	**41.5031**	**0.9879**
Sample rate: 20%	41.4073	0.9852	41.0897	0.9866	42.8114	0.9910	**43.2453**	**0.9918**
Sample rate: 30%	44.1260	0.9931	44.0760	0.9929	44.5119	0.9944	**45.3533**	**0.9949**

*The bold value means that the experimental result value is the best*.

## Discussion

The main purpose of this study is to accurately reconstruct clear MR images from under-sampled MRI k-space data, thereby accelerating MR imaging. The experimental results have demonstrated that the proposed SARA-GAN method can obtain high-quality reconstructed MRI, even in the presence of noise. In the SARA-GAN method, we propose to use the relative average discriminator instead of the original discriminator, and the self-attention mechanism to achieve global reference. Compared with the other state-of-the-art GAN-based MRI reconstruction methods, such as DAGAN, DAWGAN, DAWGAN-GP, our SARA-GAN method can provide outstanding reconstruction performance and generate MRI images with a stronger integrity, more details, and higher evaluation indices.

The convolution operation on CNN can only work in the local domain of the convolution kernel, which makes the network miss a lot of global information. The self-attention mechanism is proposed to solve the above problem by capturing long-range interactions. In this study, we apply the self-attention mechanism in the up-sampling block of the generator to combine local and global spatial information. To evaluate the impact of the self-attention mechanism on network reconstruction, we removed the self-attention layer in the up-sampling block of the generator and conducted training and testing under the same experimental conditions. The average PSNR and SSIM of the test set are shown in Table 4. As can be seen from the table, in all under-sampling modes, the self-attention mechanism affects improving the quality of reconstructed MRI images. The average PSNR is improved 0.32 ~ 1.03 dB and the corresponding SSIM improvements are 0.0005 ~ 0.0027.

**Table 4:** The influence of self-attention mechanism and SN on PSNR and SSIM of the reconstructed image.

	**RA-GAN**		**SARA-GAN(NoSN)**		**SARA-GAN**	
**Mask: 1D Gaussian**	**PSNR**	**SSIM**	**PSNR**	**SSIM**	**PSNR**	**SSIM**
Sample rate: 10%	35.9669	0.9686	35.6708	0.9669	**36.3926**	**0.9713**
Sample rate: 20%	42.8805	0.9924	42.9769	0.9925	**43.2054**	**0.9929**
Sample rate: 30%	42.7548	0.9920	43.1507	0.9927	**43.3522**	**0.9931**
**Mask: 2D Gaussian**	**PSNR**	**SSIM**	**PSNR**	**SSIM**	**PSNR**	**SSIM**
Sample rate: 10%	41.1679	0.9867	40.5628	0.9848	**41.6323**	**0.9881**
Sample rate: 20%	43.0552	0.9912	42.1689	0.9894	**43.4991**	**0.9920**
Sample rate: 30%	44.7239	0.9940	44.3378	0.9932	**45.7536**	**0.9951**

*The bold value means that the experimental result value is the best*.

**Figure 7:**
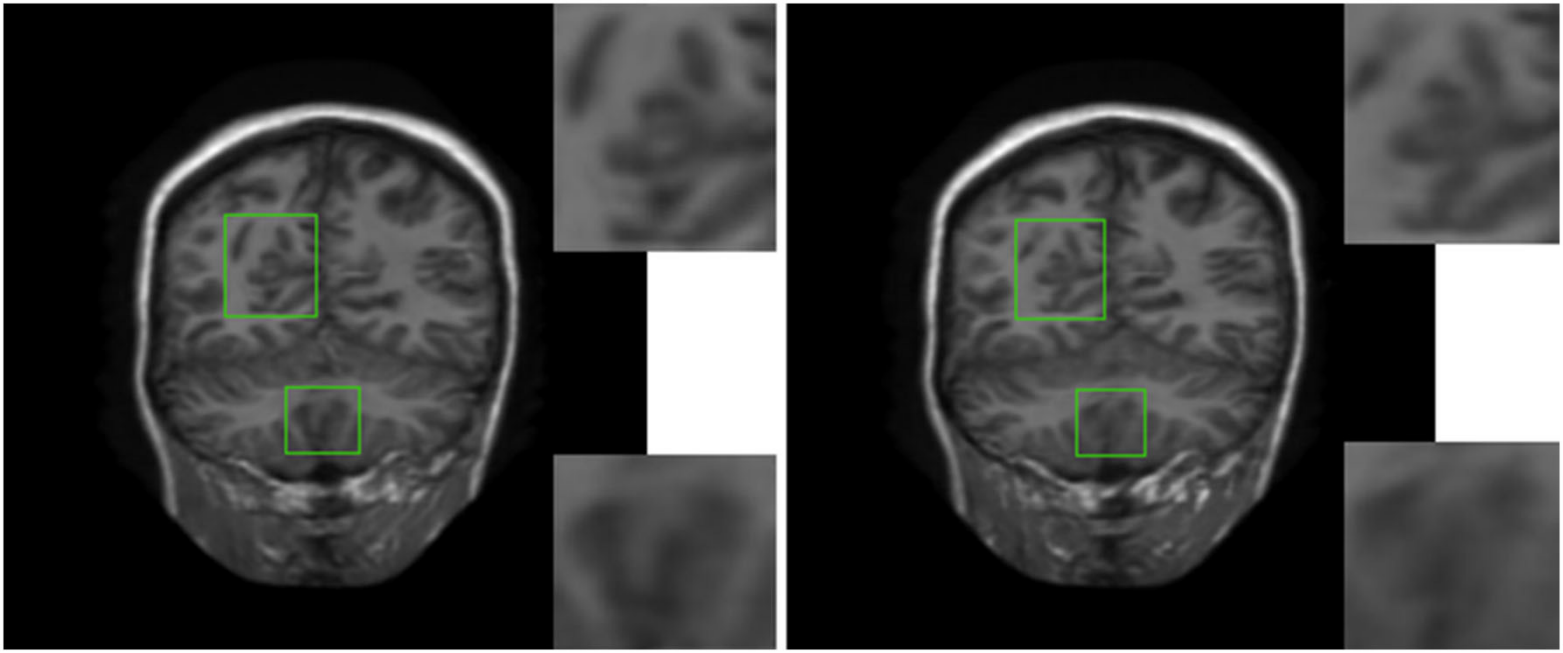
The visual influence of self-attention mechanism on the reconstructed image. Proposed RASA-GAN (PSNR: 33.5931) and Proposed (NoSA) RA-GAN (PSNR: 32.9152).

In order to verify the visual effect of the long-range dependence constructed by the self-attention mechanism on the reconstructed MRI, we selected a typical MRI and enlarged the texture-rich regions locally, as shown in Figure 7. Observation shows that the brain texture in the left picture is rich in detail and structural information is relatively complete. Comparing the enlarged image of the same area, the left image has a clear texture boundary and relatively complete color blocks, while the right image has blurry borders, and the color blocks are somewhat broken. Therefore, under the action of the self-attention mechanism, the integrity of reconstructed MRI is stronger and the visual effect is improved.

We also apply spectral normalization to the parameter matrix of the generator and discriminator.

**Figure 8:**
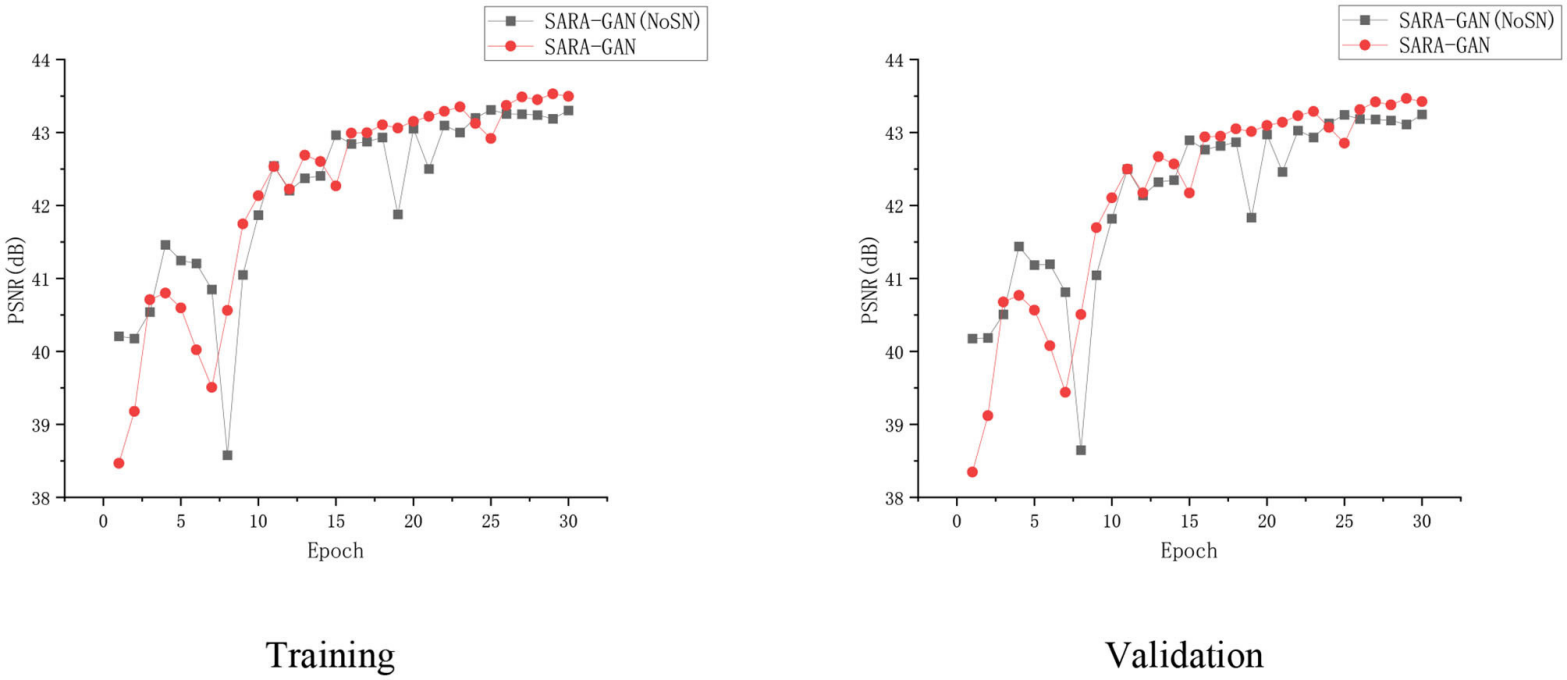
The convergence curve of the PSNR vs. the Epoch number in the case of a 30% sampling rate with the 1D Gaussian mask. Training and validation.

Spectral normalization makes the parameter matrix meet 1-Lipschitz continuity by applying the spectral norm to the network parameters, which limits the network gradient change, thereby making the training process more stable. We have conducted the convergence analyses in every epoch by using SARA-GAN and SARA-GAN without SN methods in the case of the 30% sampling rate with a 1D Gaussian mask. As shown in Figure 8, the convergence of SARA-GAN method is more stable than SARA-GAN without the SN method. Table 4 also shows the experimental results of SARA-GAN without the SN method on the test set. It can be seen that SN significantly improves the quality of network reconstruction MRI. Under the same number of iterations, due to the improvement of training stability, the method with SN can achieve a more optimized state.

## Conclusion

In this study, a new MRI reconstruction method, named SARA-GAN, was proposed to reduce k-space sampling and accelerate MRI imaging. Our method combines the self-attention mechanism with relative average discriminator. Compared with other GAN-based methods, such as DAGAN, DAWGAN, and DAWGAN-GP, the experimental results show that our method can obtain more accurate reconstructed MRI with a higher PSNR and SSIM. Especially through the long-range global dependence constructed by the self-attention mechanism, the proposed method can reconstruct images with more realistic details and stronger integrity. At the same time, the proposed method has a certain ability of noise tolerance and short reconstruction time. It provides a promising approach to speed up the MRI.

## Data Availability Statement

Publicly available datasets were analyzed in this study. This data can be found here: https://mrbrains13.isi.uu.nl/data/, MICCAI 2013 grand challenge public data set.

## Author Contributions

ZY, MJ, YW, BW, and GY: conceptualization. ZY, MJ, YW, BW, YL, PW, and GY: methodology. ZY, MJ, YW, and BW: formal analysis, investigation, and writing—original draft preparation. WM-S, ZN, and GY: writing—review and editing. MJ and GY: funding acquisition. All authors contributed to the study conception, design, read, and approved the final manuscript.

## Conflict of Interest

Co-authors ZN and WM-S are employed by Aladdin Healthcare Technologies Ltd. The remaining authors declare that the research was conducted in the absence of any commercial or financial relationships that could be construed as a potential conflict of interest.
